# [Retracted] SIRT6 promotes ferroptosis and attenuates glycolysis in pancreatic cancer through regulation of the NF‑κB pathway

**DOI:** 10.3892/etm.2024.12751

**Published:** 2024-10-23

**Authors:** Shuangxi Gong, Lixin Xiong, Zhen Luo, Qinghua Yin, Ming Huang, Yang Zhou, Jian Li

Exp Ther Med 24:502, 2022; DOI: 10.3892/etm.2022.11430

Following the publication of this paper, it was drawn to the Editors’ attention by a concerned reader that the tumor images shown in Fig. 5A on p. 9 were strikingly similar to data that has already appeared in different form in another article published in the journal *Annals of Translational Medicine* that had been written by different authors at different research institutes. In view of the fact that the abovementioned data had already apparently been published previously, the Editor of *Experimental and Therapeutic Medicine* has decided that this paper should be retracted from the Journal. After having been in contact with the authors, they accepted the decision to retract the paper. The Editor apologizes to the readership for any inconvenience caused.

